# Disentangling Human Tolerance and Resistance Against HIV

**DOI:** 10.1371/journal.pbio.1001951

**Published:** 2014-09-16

**Authors:** Roland R. Regoes, Paul J. McLaren, Manuel Battegay, Enos Bernasconi, Alexandra Calmy, Huldrych F. Günthard, Matthias Hoffmann, Andri Rauch, Amalio Telenti, Jacques Fellay, the Swiss HIV Cohort Study

**Affiliations:** 1 Institute of Integrative Biology, ETH Zurich, Zurich, Switzerland; 2 Global Health Institute, EPF Lausanne, Lausanne, Switzerland; 3 Institute of Microbiology, University of Lausanne, Lausanne, Switzerland; 4 Swiss Institute of Bioinformatics, Lausanne, Switzerland; 5 Division of Infectious Diseases and Hospital Epidemiology, University Hospital Basel, University of Basel, Basel, Switzerland; 6 Division of Infectious Diseases, Regional Hospital Lugano, Lugano, Switzerland; 7 Geneva University Hospital, HIV Unit, Department of Internal Medicine, Geneva, Switzerland; 8 Division of Infectious Diseases and Hospital Epidemiology, University Hospital Zurich, University of Zurich, Zurich, Switzerland; 9 Division of Infectious Diseases and Hospital Epidemiology, Cantonal Hospital St.Gallen, St.Gallen, Switzerland; 10 University Clinic of Infectious Diseases, University Hospital Bern and University of Bern, Bern, Switzerland; Stanford University, United States of America

## Abstract

In ecology, “disease tolerance” is defined as an evolutionary strategy of hosts against pathogens, characterized by reduced or absent pathogenesis despite high pathogen load. To our knowledge, tolerance has to date not been quantified and disentangled from host resistance to disease in any clinically relevant human infection. Using data from the Swiss HIV Cohort Study, we investigated if there is variation in tolerance to HIV in humans and if this variation is associated with polymorphisms in the human genome. In particular, we tested for associations between tolerance and alleles of the Human Leukocyte Antigen (HLA) genes, the CC chemokine receptor 5 (CCR5), the age at which individuals were infected, and their sex. We found that *HLA-B* alleles associated with better HIV control do not confer tolerance. The slower disease progression associated with these alleles can be fully attributed to the extent of viral load reduction in carriers. However, we observed that tolerance significantly varies across *HLA-B* genotypes with a relative standard deviation of 34%. Furthermore, we found that *HLA-B* homozygotes are less tolerant than heterozygotes. Lastly, tolerance was observed to decrease with age, resulting in a 1.7-fold difference in disease progression between 20 and 60-y-old individuals with the same viral load. Thus, disease tolerance is a feature of infection with HIV, and the identification of the mechanisms involved may pave the way to a better understanding of pathogenesis.

## Abstract 2

Title: Human tolerance against HIV An evolutionary ecology perspective on clinical data reveals that human traits can affect how well an individual tolerates HIV infection, and identifies host immunity factors associated with disease tolerance.

## Author Summary

When confronted with pathogens, hosts can either evolve to fight them or learn to live with them. The first of these two strategies is called “resistance” and the second “tolerance”. In the context of HIV, many genes conferring resistance have been identified, but no tolerance genes are known. Using statistical techniques originating from plant ecology, we analyzed data from an HIV cohort to look for differences in tolerance between HIV-infected individuals and tested whether they go hand in hand with genetic differences. We found that younger people are more tolerant to HIV infection. We also observed that individuals who carry two different alleles of HLA-B, an important immunity gene, are more tolerant. These findings add to our understanding of how hosts tolerate infections and could open new avenues for treating infections.

## Introduction

In response to pressure by pathogens, host populations can evolve in two ways: They can develop either resistance or tolerance to the disease [1]–[8]. Resistance mechanisms reduce the pathogen burden. Tolerance mechanisms, in contrast, reduce the damage that accompanies infection without affecting the pathogen directly. One of the best examples for tolerance are sooty mangabeys infected with Simian Immunodeficiency Virus (SIV), which—despite harboring high virus loads—do not develop disease [9].

Whether hosts evolve resistance or tolerance affects the evolutionary trajectory of host-pathogen systems [2],[3],[10]–[12]. The evolution of resistance genes in the host provokes counteradaptations of the pathogen that overcome host resistance, resulting in an endless arms race. In contrast, tolerance genes benefit both the host and the pathogen and are therefore predicted to fix.

It is increasingly recognized that disentangling resistance and tolerance not only advances our understanding of the coevolution between hosts and pathogens but also is relevant clinically [13]. Like resistance factors, mechanisms of tolerance, once identified, can be exploited for therapy. In contrast to resistance-based therapy, tolerance-based treatment does not aim at reducing the pathogen load but rather at ensuring the well-being of the host. For that reason, tolerance-based therapy is also hypothesized to be evolution-proof—that is, not to select for drug-resistant pathogens [4],[5],[14]. It has been argued, however, that the pathogen population might evolve higher virulence in response to tolerance-based treatment [3],[15],[16].

Although numerous review papers have been written on the potential benefits of tolerance research [1]–[8], the formal framework for disentangling tolerance and resistance has not been applied to many animal disease systems. There is a paradigmatic study on mouse malaria [17] and a few on insects [18]–[20]. But a quantitative tolerance analysis has, to our knowledge, not yet been conducted for any clinically relevant human disease. In this study, we apply such an analysis to HIV infection in humans.

**Figure 1:**
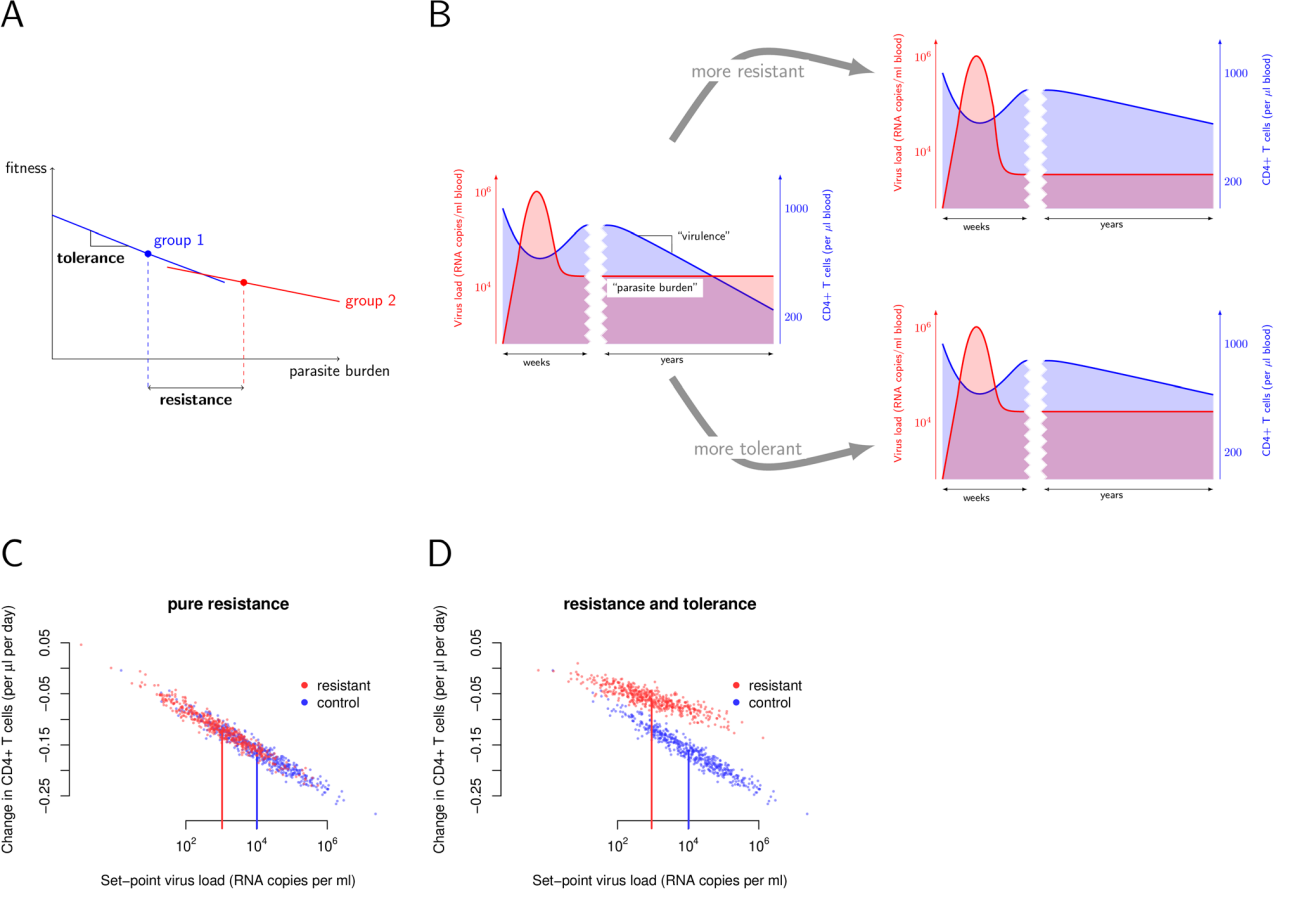
Quantifying tolerance and resistance. (A) The tolerance of a group of individuals can be measured as the change of fitness across varying levels of parasite burden. Fitness is inversely related to the virulence of the infection. The difference in resistance between groups can be quantified simply as the difference in the mean parasite burden. (B) In the context of HIV, virulence can be quantified by measuring the CD4+ T-cell decline in an infected individual, and the set-point viral load is a good proxy for the “parasite burden”. (C) and (D) show conceivable outcomes of a tolerance-resistance analysis for the HIV resistance genes, such as classic protective *HLA-B* alleles. In the scenario entitled “pure resistance” (C), the reduction of viral load that the resistance genes confers fully explains the reduction in disease progression. Alternatively, resistance genes could additionally confer tolerance, as shown in plot (D).

**Figure 2:**
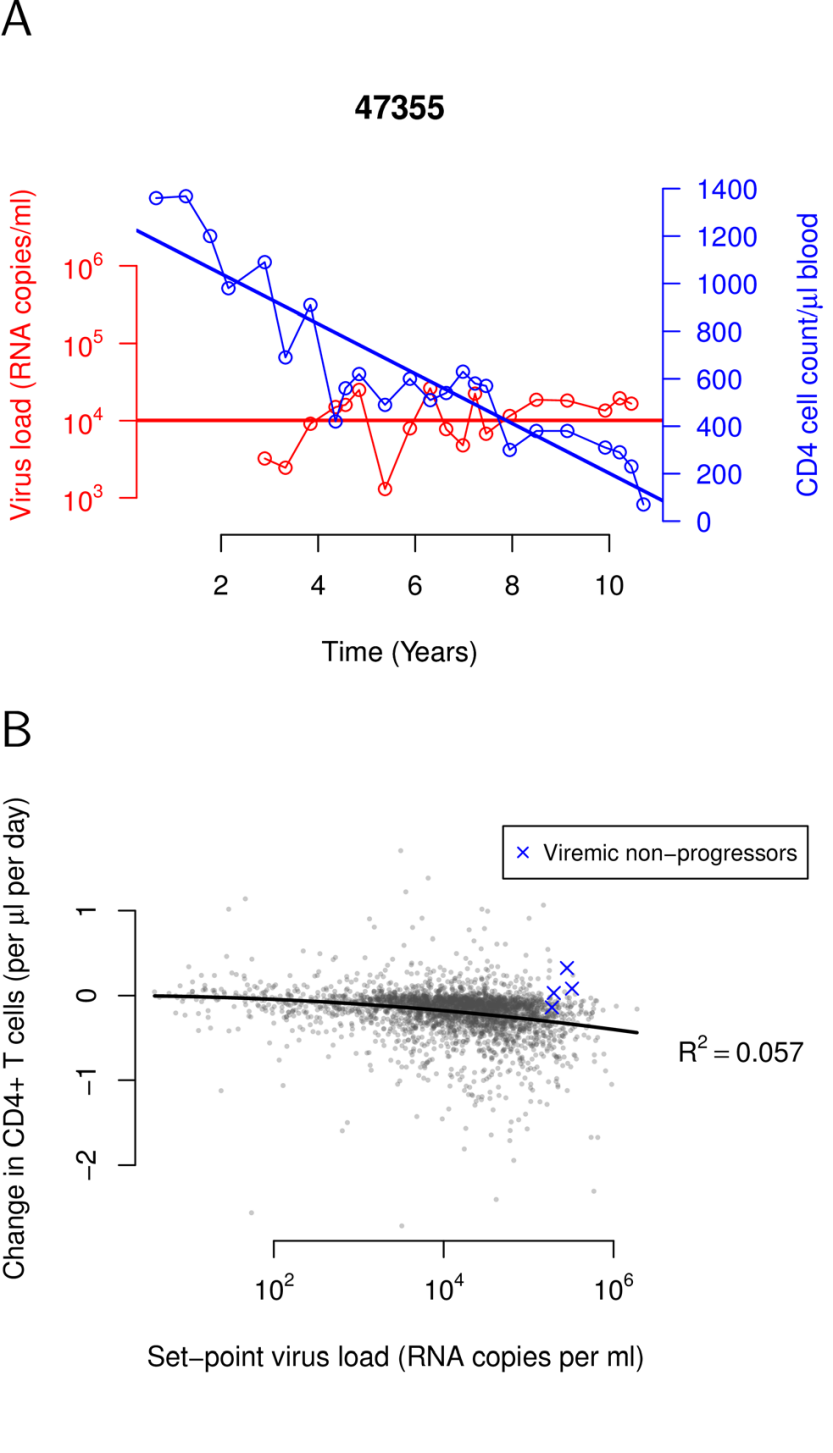
Relationship between CD4+ T-cell decline and set-point viral load in our study population. (A) Calculation of the set-point viral load and CD4+ T-cell decline, illustrated for a single individual. The set-point viral load (red line) is calculated as the geometric mean of the viral load measurements (after primary infection and before treatment). The decline of CD4+ T cells is determined as the regression slope (blue line) of CD4+ T-cell counts against time. The CD4+ T-cell counts and virus load measurements of three randomly selected individuals are shown in Figure S1. (B) Nonlinear tolerance curve characterizing the relationship between CD4+ T-cell decline and set-point viral load in our study population (*n* = 3,036). The black line shows the quadratic regression line. Blue crosses indicate individuals that were identified as viremic nonprogressors in a previous study [26].

Formally, tolerance can be quantified as the change in disease progression across different levels of pathogen burden (see Figure 1A) [2],[4]. In the context of HIV, excellent measures of disease progression and pathogen burden are available (see Figures 1B and 2A). A few weeks after infection, HIV attains a level in the plasma of infected individuals that is approximately stable over several years. This level, called the set-point viral load, is very well suited as a proxy for the “parasite burden” necessary for a formal tolerance analysis.

The rate of disease progression—the second essential parameter for an analysis of tolerance—can be measured quantitatively by the decline of CD4+ T lymphocytes. Before infection, individuals have on average 1,000 CD4+ T cells per µl of blood. A decline of CD4+ T cells below 200 per µl of blood defines AIDS. Thus, the decline of CD4+ T cells reflects what we know about the mechanistic basis of the disease. CD4+ T-cell declines have also been found to be independent predictors of disease progression in the Swiss HIV Cohort [21] that we analyzed here and other cohorts [22]. Importantly, the rate of decline can be calculated in a much shorter time scale than the direct observation of disease progression requires. The faster the CD4+ T cells decline, the higher the rate of progression toward disease and death—that is, the higher the virulence of the infection in the sense of evolutionary ecology. For these reasons, also previous studies on virulence relied on the CD4+ T-cell decline [23]. To our knowledge, such a well-established, quantitative measure of virulence is not available for any other human infection.

## Results

We determined set-point viral loads and CD4+ T cell declines in 3,036 HIV-1–infected individuals (see Figure 2, Materials and Methods, and Data S1). To investigate tolerance of humans against HIV, we determined the relationship between CD4+ T-cell decline and set-point viral load in our study population. We started by establishing this relation for the entire study population. In subsequent analyses, this relationship served as a baseline, against which we later compared the relationships between CD4+ T-cell decline and set-point viral load in specific subgroups. Finally, we used the baseline relationship to define a tolerance phenotype for each individual in our study population and investigated if they are associated with single nucleotide polymorphisms (SNPs) in the human genome.

### Tolerance Curve Is Nonlinear

To establish the baseline relationship between CD4+ T-cell decline and viral load, we performed a regression analysis. We found that this relationship is significantly nonlinear (see Figure 2). Although nonlinear tolerance curves are a departure from what has been reported in other systems, this finding is not surprising. Linearity is an assumption generally adopted in regression analyses mostly for the sake of simplicity and convenience. Commonly, low sample sizes precluded the assessment of a potential nonlinearity. The establishment of such a nonlinearity in the context of tolerance, however, is particularly crucial to reliably establish tolerance differences between groups [24].

The relationship is best described by a quadratic relationship (see Figure 2B and Text S1). The intercept of the relationship is not significantly different from 0. This is in line with the expectation that uninfected individuals should have relatively stable CD4+ T-cell counts. Also the linear term is not significantly different from 0.

Mathematically, we can write the relationship as:



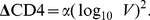
(1)


In this equation, 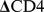 denotes the rate of change of CD4+ T cells per µl of blood per day, and 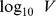 the logarithm to the base 10 of the viral load per ml of plasma. The quadratic model explains 5% of the variation in CD4+ T-cell decline, consistent with previous studies investigating this relationship with linear models [25].

The parameter *α* is the quantitative measure of the average tolerance across the entire study population, which we used in the present study. It describes how the relationship curves downwards; that is, it measures how the decline in CD4+ T cells, 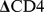
—a surrogate measure of disease progression—changes with the set-point viral load. For a value *α* = 0, CD4+ T cells would not decline irrespective of the set-point viral load. This case would correspond to complete tolerance. If *α*<0, an increase in the set-point viral load accelerates the progression towards disease. The lower *α*, the lower the tolerance. For the entire study population, we estimated *α* = −0.0111±0.0003.

Four individuals with an infection characterized by very high viral load and minimal disease progression are also depicted in Figure 2B. They lie above the average tolerance curve. These individuals, referred to as viremic nonprogressors [26], share the transcriptomic, interferon response, and gut microbial translocation profile of nonpathogenic SIV infection in their natural host species [26]–[28]. Thus, the tolerance analysis correctly identified individuals whose tolerance had been previously established.

### Tolerance, Sex, and Age

**Figure 3:**
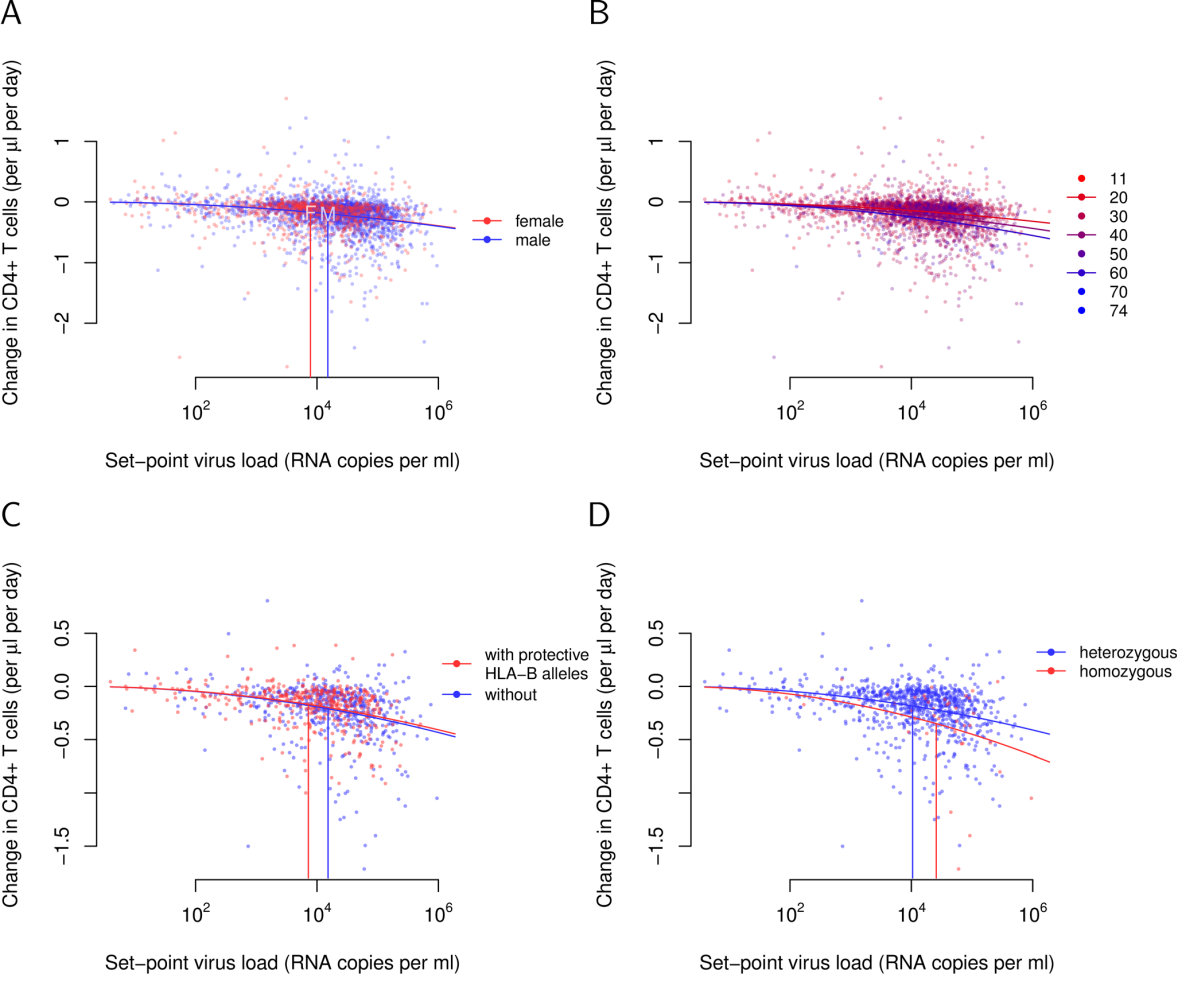
Investigating associations of tolerance with sex, age at infection, and HLA-B alleles. (A) Tolerance does not differ significantly between sexes in a univariate analysis. (B) Young age at infection is strongly associated with tolerance. The data are plotted stratified by age. The younger, the redder. The three curves show the relationships between set-point viral load and CD4+ T-cell decline when infected at age 20, 40, and 60. (C) Classic protective *HLA-B* alleles induce pure resistance. The tolerance curves do not differ significantly for individuals with (red, *n* = 416) and without (blue, *n* = 507) protective *HLA-B* alleles. Protectiveness is defined according to the data presented in table 1 of [30] (see Materials and Methods). (D) *HLA-B* homozygosity is associated with tolerance. Homozygotes also have significantly higher set-point viral loads—that is, are more resistant than heterozygotes.

First we tested if the tolerance parameter differs with sex and the age at which individuals were infected. Information on these demographic characteristics was available for all 3,036 individuals in our study population (see Materials and Methods). Although females had an almost 2-fold lower viral load set-point than males, we did not find significant differences in tolerance between sexes, either in a univariate analysis (*F* test: *p* = 0.69; Figure 3A) or in an analysis adjusting for age difference between sexes (*F* test: *p* = 0.45). This result challenges previous reports, according to which females are less tolerant (see Discussion) [29].

The age at which individuals become infected with HIV, however, was very strongly associated with tolerance (Figure 3B), both in univariate (*F* test: *p* = 10^−9^) and multivariate analyses controlling for sex (*F* test: *p*<3×10^−8^). According to this analysis, at equivalent viral load, the disease progression rate of an individual who contracts HIV at the age of 60 is 1.7-fold faster than that of an individual becoming infected at the age of 20.

### No Association of Tolerance with Known Resistance Genes

Next, we investigated if the tolerance parameter *α* differs across well-established human genetic polymorphisms associated with HIV control and disease progression—that is, resistance to HIV in the sense of evolutionary ecology. For more than 850 individuals in our study population, information on *HLA* class I alleles and the CC chemokine receptor 5 (*CCR5*) genotype was available (see Materials and Methods).

In a first step, we focused on *HLA-B* alleles that have been found to associate with lower viral load—that is, with resistance [30]. We wondered if these alleles are also associated with tolerance. We found that protective *HLA-B* alleles are not associated with higher or lower tolerance in a univariate analysis (*F* test: *p* = 0.40; Figure 3C). This is independent of how stringently we define protective *HLA-B* alleles (see Materials and Methods and Figure S2). Thus, the protection these alleles confer can be fully attributed to the effect they have on viral load.

Higher *HLA-C* expression has been associated with better control of HIV viremia and slower disease progression [31]–[33]. The expression level of *HLA-C* is reasonably predicted by classical *HLA-C* alleles, which are in strong linkage disequilibrium with a causal polymorphism in the 3' untranslated region of *HLA-C* [33]. We could thus predict the *HLA-C* expression level for 850 individuals in our study population, of which 243, 434, and 173 had low, medium, and high expression, respectively. We found that the tolerance parameter *α* does not vary significantly with *HLA-C* expression in a univariate analysis. We also did not find any association of tolerance with protective *HLA-B* alleles and predicted *HLA-C* expression in a multivariate analysis including both factors together with sex and age at infection as covariates.

Another important polymorphism related to HIV acquisition and disease progression is located in the gene coding for the chemokine receptor *CCR5*. About 10% of Europeans carry a *CCR5* allele with a 32 base pair deletion (*CCR5*Δ32). Homozygous individuals are almost completely resistant to infection, while carriage of a single allele has been reported to be associated with slightly lower set-point viral load and slower disease progression [34]. We divided the fraction of our study population, for which we had information on the *CCR5* genotype, into individuals with (*n* = 163, all heterozygous) and without (*n* = 699) *CCR5*Δ32. There was no significant difference in tolerance between these two groups in a univariate analysis. Again, we obtained the same result in a multivariate analysis including sex and age at infection as covariates.

### Variation of Tolerance Associated with *HLA-B* Combinations

The analyses above aimed at determining if known resistance genes also induce tolerance. We found that they do not. But what if there are yet unknown genes, unrelated to resistance, that confer tolerance?

**Figure 4:**
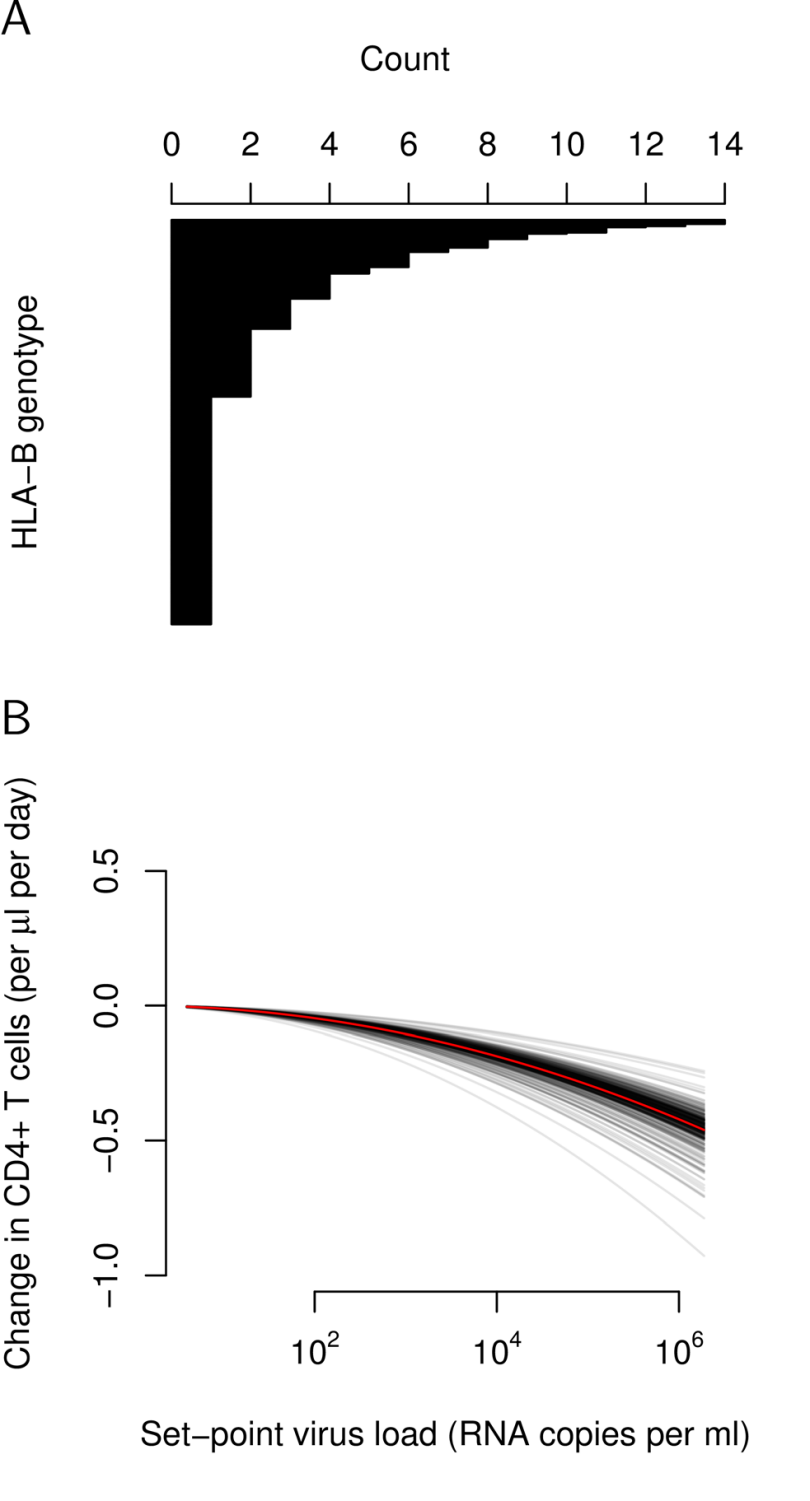
Variation of tolerance associated with *HLA-B* genotype. (A) Frequencies of the *HLA-B* genotypes in our study population of 923 individuals. Approximately half of the genotypes are represented by only one individual. (B) Visualizing the random effect of the mixed effect modeling approach. Estimated tolerance curves for each *HLA-B* genotype, based on best linear unbiased predictions, are shown. We estimated a mean tolerance parameter 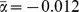 (red curve), and a deviation of the random effects, 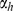, of 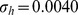 (see Text S1).

As first candidates for such tolerance genes, we considered *HLA-B* alleles irrespective of their protectiveness. To assess if there are differences in tolerance associated with *HLA-B*, we adopted a mixed-effects modeling approach. We combined the two *HLA-B* alleles of an individual into a genotype (see Materials and Methods) obtaining 375 unique genotypes in our study population. The frequency distribution of the combined *HLA-B* genotypes is shown in Figure 4A.

In the mixed-effects models, we used *HLA-B* genotype as a random effect. Specifically, we assumed the following relationship between CD4+ T-cell decline, ΔCD4, and set-point viral load, *V*, in a univariate analysis:



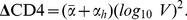
(2)


The parameter 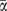 characterizes the average tolerance in our study population, and 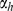 denotes how the tolerance of genotype *h* deviates from this average. We treated this parameter as a random effect—which means that we did not estimate it for each genotype but estimated the variance of its distribution (see Text S1).

We found significant variation in the random effect *α_h_* of *HLA-B* genotypes. Compared to a model without this random effect with a likelihood ratio test, we obtained a significance level of *p* = 0.0002. This variance is illustrated in Figure 4B: across *HLA-B* genotypes, tolerance differs approximately 2-fold and the relative standard deviation (the standard deviation divided by the absolute value of the mean) is 0.34. This variance in tolerances translates into an approximately 1.7-fold difference in the rate of disease progression for two randomly selected HLA-B genotype groups. Restricting our analysis to genotypes represented by more than one individual yields an even larger and more significant random effect, and a multivariate analysis that includes sex and age at infection as covariates shows that these two variables do not confound our analysis (see Text S1).

Table 1 lists 5% (*n* = 18) of the *HLA-B* genotypes with the most extreme tolerance as predicted by the mixed-effects model. The values in Table 1 are best linear unbiased predictions [35], rather than estimates of tolerance parameters for each combined *HLA-B* genotype group, and should therefore be interpreted with care. Figure S3 shows a histogram of the best linear unbiased predictions of tolerance for the *HLA-B* genotypes.

**Table 1:** The nine most and the nine least tolerant *HLA-B* genotypes.

*HLA-B* Genotype	Tolerance^a^	Frequency^b^
0702/3901	−0.0061 (most tolerant)	4
1501/3906	−0.0063	1
1801/4403	−0.0067	7
5301/5801	−0.0077	2
1501/5001	−0.0080	1
1801/5101	−0.0082	10
4402/4402	−0.0082	3
1801/4402	−0.0089	4
3501/4501	−0.0089	1
4002/4501	−0.0164	1
1801/2705	−0.0169	7
4403/4403	−0.0172	1
1402/5001	−0.0174	2
1801/4002	−0.0179	1
4402/5001	−0.0180	3
3503/5101	−0.0180	7
1402/4403	−0.0200	5
3501/3501	−0.0235 (least tolerant)	3

a Best linear unbiased predictions of the tolerance parameter 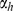 for each genotype.

b Number of individuals with the respective genotype among the 923 individuals studied.

As outlined in Text S1, we could not identify any association of tolerance with particular *HLA-B* alleles, suggesting that the effects of the two *HLA-B* alleles on tolerance depend on the specific combination of *HLA-B* alleles, rather than just on the sum of their effect (see Figure S4). A case in point is the least tolerant genotype group “3501/3501”. Carriage of this allele (considering homo- and heterozygotes together) is not associated with higher set-point virus load, faster CD4+ T-cell decline, or lower tolerance. But HLA-B*3501 homozygotes display the most extreme departure from the average tolerance curve. This is due to a very fast CD4+ T-cell decline in two individuals in this genotype group.

### *HLA-B* Homozygosity Is Associated with Lower Tolerance

To further explore the importance of *HLA-B* allele combination on tolerance, we compared homozygous to heterozygous individuals. Of the 923 individuals in our study population, for which we have information on the *HLA-B* alleles they carry, 39 were homozygous, displaying 14 unique genotypes. A regression analysis of the CD4+ T-cell decline against set-point viral load with *HLA-B* homozygosity as a covariate confirmed a significant association of homozygosity with tolerance in univariate (*F* test: *p* = 0.00016) and multivariate analysis including sex and age at infection (*F* test: *p* = 0.00005).

Figure 3D depicts the difference in tolerance between hetero- and homozygotes according to a univariate analysis. Homozygotes have higher set-point viral loads than heterozygotes and are therefore expected to display faster CD4+ T-cell declines. Figure 3D, however, shows that the CD4+ T-cell decline is in fact much faster in homozygotes than their set-point viral load predicts. Quantitatively, the tolerance paramete *α* of homozygotes is −0.019 (versus *α* = −0.012 in heterozygotes). This difference in the tolerance parameter translates into a 1.6-fold faster rate of disease progression of homozygotes compared to heterozygotes with the same set-point viral load. The tolerance difference between homo- and heterozygotes further supports the view that the effect of *HLA-B* alleles is not additive and refines our understanding of the well-established *HLA*-heterozygote advantage with respect to set-point virus load and disease progression [36],[37].

### No Trade-Off Between Tolerance and Resistance

In contrast to previous studies on tolerance and resistance [17], we did not find a trade-off—that is, a negative correlation—between resistance and tolerance across *HLA-B* genotype groups (see Text S1). The lack of a correlation between tolerance and resistance suggests that there are no mechanistic or genetic constraints to display both traits. If both tolerance and resistance mechanisms are costly, a trade-off could eventually evolve, but the co-evolutionary history between humans and HIV may have been too short for distinct resistant and tolerant lineages to separate. However, we found a positive relation between tolerance and resistance across age. As individuals get older they become less tolerant and less resistant.

### No Genome-Wide Association with Tolerance

We also looked for genome-wide associations with tolerance. To this end, we defined a tolerance phenotype for each individual by calculating the residual in a quadratic regression between an individual's CD4+ T-cell decline and viral load, controlling for the age at infection (see Figure S5A). This analysis failed to identify any SNPs associated with tolerance (Figure S5B). It is important to note that this analysis, in addition to setting very stringent requirements for significance by correcting for multiple testing, also assumes additivity of allelic effects—that is, ignores a potential heterozygote advantage.

## Discussion

In summary, we presented the first formal tolerance analysis of a clinically relevant human infection. HIV infection features well-established measures of pathogen burden and disease progression that are required for such an analysis. The analysis consistently identified a subset of individuals that tolerate high viral load with minimal disease progression—the so-called viremic nonprogressors [26], whose biological profile (transcriptome, interferon response, gut microbial translocation) is reminiscent of SIV infection in sooty mangabeys [26]–[28].

But beyond this consistency with the tolerant profile of these four individuals, adopting the evolutionary ecology framework for tolerance allowed us to assign quantitative tolerance measures to well-defined groups of individuals and to statistically compare them. In addition to investigating age- and sex-related differences in tolerance to HIV, we could, due to the wealth of information available for individuals in the Swiss HIV Cohort Study, test for potential associations with genes implicated in disease susceptibility and progression, such as *HLA* class I and *CCR5*.

The finding that there is no difference in tolerance between the sexes challenges a previous report by Farzadegan et al. [29], according to which females are less tolerant than males. Just like Farzadegan et al., we found that females have significantly lower viral loads, but do not differ in their disease progression. In contrast to Farzadegan et al., however, this pattern did not result in a significant difference in the relationship between disease progression and set-point viral load. One reason for this discrepancy may be that Farzadegan et al. used data on AIDS diagnosis during a time window of observation, whereas we used CD4+ T-cell decline to measure disease progression. Furthermore, Farzadegan et al. performed a survival analysis, whereas we performed a regression analysis. Lastly, in contrast to our analysis, Farzadegan et al. did not adjust for the age at which individuals became infected. For all these reasons, the previous and present analyses are difficult to compare and the discrepancy remains unresolved.

In all of the figures that show our data, it is apparent that the relationship between the set-point viral load and CD4+ T-cell decline is weak. The noise in this relation is entirely consistent with previous studies [25] in which 5%–9% of the variation in the CD4+ T-cell decline could be explained by the set-point viral load. The analysis we performed to identify variation in tolerance aimed at detecting differences in this relationship between different subgroups in our study population. Given how noisy this relation is, it is remarkable that we could identify significant associations of host factors with tolerance at all.

In our study, we considered the most important host genes but disregarded the potential impact of virus genetics on tolerance. The viruses harbored by the individuals in our study population differ by subtype. Although viral subtypes are hypothesized to vary in virulence, this effect is difficult to ascertain due to usually unaccounted differences in the study populations [38]. However, a large fraction of individuals in the Swiss HIV Cohort carry subtype B virus [39],[40]. We therefore do not expect the genetic variation of the virus to confound our analysis.

The framework for investigating tolerance we adopted for this study, despite its internal consistency, has its limits. The parasite burden—central as the x-axis in our tolerance curve plots—is not simply an external factor affecting virulence but will itself be influenced by the host genotype and phenotype. If we had virus dynamics models that described the entire course of HIV infection, the relationship between virulence and virus load could be mechanistically derived, and we would not have to rely on the statistical approach adopted here. Such a comprehensive model has, however, been elusive to date [41], mostly because the slow depletion of CD4+ T cells cannot be accounted for by HIV targeting and killing these cells. Rather, a generalized immune activation in infected individuals is currently conceived to be at the heart of the mechanisms of pathogenesis [42], and a straight-forward relationship between set-point virus load and CD4+ T-cell decline is unlikely to emerge from the probably complex dynamics. Until a better dynamical understanding of HIV pathogenesis emerges, the low power of the set-point virus load to predict the CD4+ T decline [25] provides some justification of treating these two entities as independent.

Our analysis implicates *HLA-B* in modulating tolerance. In particular, we established a tolerance advantage of *HLA-B* heterozygotes, providing an additional example of a benefit that host diversity affords against pathogens [36],[43]–[46]. Mechanistically, it is conceivable that certain *HLA-B* alleles cause faster disease progression without increasing viral load by modulating immunopathology, rather than leading to the killing of infected cells by cytotoxicity. The higher tolerance of individuals, who contracted HIV at a young age, is likely to be explained by the higher thymic output of young individuals that can compensate infection-related CD4+ T-cell loss [47]. Confirming or refuting these hypothetical mechanisms will be an important direction of future research on tolerance against HIV.

## Materials and Methods

### Ethics Statement

The Swiss HIV Cohort Study was approved by the local Ethics Committees of all participating centers, and written informed consent was obtained from the participants. This project was approved by the Scientific Board of the SHCS as project 697.

### Study Population

We used data from the Swiss HIV Cohort Study (www.shcs.ch) [48]. Briefly, the study has enrolled more than 18,000 HIV-infected individuals to date. Sociodemographic and behavioral data are recorded at entry to the study, in particular year of birth, gender, and the date of the last negative HIV test. Laboratory and clinical data, including viral load and CD4+ T-cell count, are obtained at each semiannual follow-up visit. Approximately 2,000 individuals have been genotyped in the context of previous genome-wide association studies [31],[49] and/or at loci relevant for HIV acquisition and disease progression, such as those encoding the Human Leukocyte Antigen (HLA) class I genes and *CCR5*.

We included individuals into our study, for whom viral load measurements and CD4+ T-cell counts were available, to reliably estimate the set-point viral load and CD4+ T-cell decline, as defined below. We restricted our analysis to data obtained before antiretroviral treatment because the relationship between CD4+ T-cell count and viral load is dramatically altered during treatment. To exclude the primary infection period, during which viral load and CD4+ T-cell count exhibit strong fluctuations, we discarded results obtained during the first 90 days after the estimated date of infection. To exclude the late phase of the infection, during which viral load increases and fluctuates due to severe immunosuppression, we discarded measurements obtained when the CD4+ T-cell count was below 100 per µl. Individuals were included if they had at least two eligible viral load results and three eligible CD4+ T-cell measurements at least 180 days apart.

After applying these inclusion criteria, our study population comprised 3,036 individuals. For 837, 923, and 862 individuals, we had information on the *HLA-A*, *-B*, and *-C* alleles, respectively. The *CCR5*Δ32 genotype was available for 862 individuals, whereas 852 individuals had genome-wide genotyping results. Of the 923 individuals, for whom we had information on the *HLA-B* alleles, a large majority of 850 were of European ancestry.

### Calculation of Set-Point Viral Load, CD4+ T-Cell Decline, and Definition of Subgroups

Set-point viral load was determined as the geometric mean of the eligible viral load measurements in each individual. Nondetectable viral loads were set to half the detection limit. The change of CD4+ T-cell count over time was estimated as the slope in a linear regression of CD4+ T-cell count against the date at which they were determined. Data S1 provides estimates of the set-point viral load and CD4+ T-cell declines for the 3,036 individuals included in our study.

We defined an *HLA-B* allele as “protective” if it has been found to associate with better HIV control and slower disease progression, according to table 1 of [30]. In addition, we adopted alternative, more restrictive definitions, considering either only *HLA-B*27 or 57, or only HLA-B*27:05 and *57:01 as protective (see Figure S2).

The *HLA-C* expression levels of the individuals in our study were predicted from the classical *HLA-C* alleles using data from table S1 in Kulkarni et al. [33].

For each individual, a combined *HLA-B* genotype was defined by concatenating and sorting the four-digit alleles they carry. An example for a genotype thus defined is “0702/3501”.

### Statistical Analysis

The statistical analysis is comprehensively described in Text S1. Here we just give a brief overview of the logic of our statistical procedures.

We regressed the change in CD4+ T cells over time, ΔCD4, against the set-point viral load, *V*, using a least-square fitting algorithm assuming linear and nonlinear relationships. Sex, age at infection, protectiveness of *HLA-B* alleles, carriage of *CCR5*Δ32, predicted *HLA-C* expression levels, and *HLA-B* homozygosity were included into the regression analysis as covariates either individually or in combination.

Formally, we investigated the association of tolerance with a binary factor, such as sex or the carriage of protective HLA-B alleles, by decomposing the parameter *α* in the baseline model (equation 1):



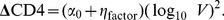
(3)


Hereby, 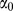 denotes the tolerance parameter for the subpopulation without the factor, and 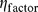 an offset associated with the factor. Multiple factors were included into the statistical model by further decomposing the tolerance parameter: 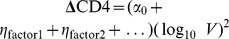.

If a factor had more than two levels, one level was defined as the baseline and an offset parameter was added for each alternative level. This was the case for *HLA-C* expression, which can be expressed at low, medium, and high levels. Consequently, the models including *HLA-C* expression as a covariate feature two offset parameters (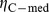 and 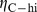
—see Text S1). Age at infection, *a*, being a continuous variable, was assumed to affect the tolerance parameter linearly:



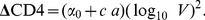
(4)


In this expression, 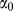 denotes the tolerance when contracting HIV at age 0, and *c* describes the increase or decrease of tolerance per life year.

We assessed if a covariate significantly affected tolerance in two ways. First, we checked if the offset associated with the covariate was significantly different from zero. Second, we compared the models with and without the covariate with an *F* test or a likelihood ratio test. In all cases, these two tests agreed. Each factor was considered on its own in a univariate analysis and in combination with the other factors in multivariate analyses (see Text S1).

The coefficient of determination of a model, *R* ^2^, was calculated as one minus the ratio between the variance of residuals in the respective model fit and the variance in ΔCD4 [50]. Note that, because our models set the intercept to zero, the variance in ΔCD4 does not represent the residual sum of squares of any special cases of our models—that is, of any model nested in our models.

### Implementation

The inclusion criteria, calculation of set-point viral load and CD4+ T-cell decline, as well as the model fitting and comparisons were implemented and performed in the R language of statistical computing [51]. Regression analysis was performed using the R-functions lm() and, for the mixed effects models, lme() in the R-package nlme(). The *F* tests and likelihood ratio tests were performed using the R-function anova().

### Genome-Wide Association Study

For the genome-wide association study, we assigned a tolerance phenotype to 852 individuals in our study population, for whom we had genomic information and who were of European ancestry. This phenotype was calculated as the deviation of the individual's set-point viral load and CD4+ T-cell decline from the average tolerance relationship of the population. Because the age at infection was associated very strongly with tolerance, we calculated the deviation from an age-controlled tolerance relationship (see Figure S5A).

Study participants had been genotyped in the context of previous studies [31],[49] using Illumina 550 or 1 M chips, and genome-wide SNPs were imputed using the 1000 Genomes Project CEU panel as a reference. After quality control and exclusion of nonvariable SNPs, seven million variants were available for association testing. We used linear regression to test for association between each SNP and the tolerance phenotype, including sex and the coordinates of the first five principle components of an EIGENSTRAT analysis [52] as covariates. We used Bonferroni correction to control for multiple testing (*p* threshold  = 5×10^−8^).

## Supporting Information

Figure S1 CD4+ T-cell count and virus load measurements in three randomly selected individuals from our study population. The red lines show the mean of the virus load measurements. The blue lines are the linear regression lines of CD4+ T-cell counts against time.
(TIFF)

Figure S2 Alternative sets of protective *HLA-B* alleles and tolerance. (A) Considering only *HLA-B*27 or 57 as protective, we did not find differences in tolerance between individuals with and without protective *HLA-B* alleles. (B) We reached the same conclusion if we are even more restrictive and assume only HLA-B*27:05 and *57:01 to be protective.
(TIFF)

Figure S3Distribution of the best linear unbiased predictions for the tolerance parameters, 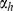, across *HLA-B* genotypes.(TIFF)

Figure S4 Tolerance by *HLA-B* allele. The tolerance parameters of genotypes containing an allele are plotted (transparent grey dots). Homozygous genotypes are plotted transparent red. Alleles are ordered by increasing mean tolerance of genotypes that contain the allele (red bars). Blue bars show the median tolerance for each allele. The variation in mean effects of each allele is significantly lower than the tolerance variation across genotypes.
(TIFF)

Figure S5 Genome-wide association study. (A) The tolerance phenotype for an individual is defined as the deviation of his/her CD4+ T-cell decline from the average tolerance curve characterizing his/her age class. Two individuals are shown (red and blue dots), together with the tolerance curves (red and blue lines) for people who contract HIV at the same age. In this example, the red and blue individuals contracted HIV at the age of 42 and 20 years, respectively. (B) Manhattan plot showing the *p* across seven million SNPs. None of the *p* is above the significance level corrected for multiple testing (dashed line).
(TIFF)

Data S1 Estimates of the set-point viral load and CD4+ T-cell decline for the 3,036 individuals in our study population.
(TSV)

Text S1 Details on the statistical analyses. This document contains a detailed description of the statistical analyses, the results of which are presented in this article. It also describes additional analyses we performed to corroborate our findings.
(PDF)

## Abbreviations

AIDS: acquired immunodeficiency syndrome
CCR5: CC chemokine receptor 5
CD4: cluster of differentiation 4
HIV: human immunodeficiency virus
HLA: human leukocyte antigen
SIV: simian immunodeficiency virus
SNP: single nucleotide polymorphism
