# Supplementary figures and images for: Disentangling Human Tolerance and Resistance Against HIV
Source: PLoS Biol. 2014 Sep 16;12(9):e1001951. doi: 10.1371/journal.pbio.1001951 (PMC4165755; doi:10.1371/journal.pbio.1001951)

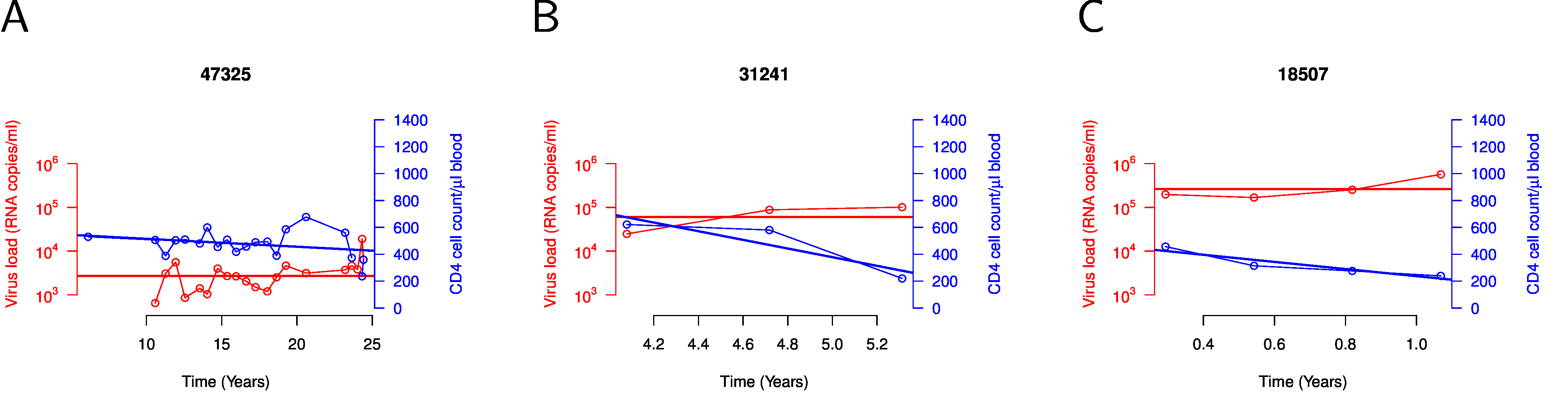

Supplement: Figure S1 — CD4+ T-cell count and virus load measurements in three randomly selected individuals from our study population. The red lines show the mean of the virus load measurements. The blue lines are the linear regression lines of CD4+ T-cell counts against time. (TIFF) [file pbio.1001951.s001.tiff]

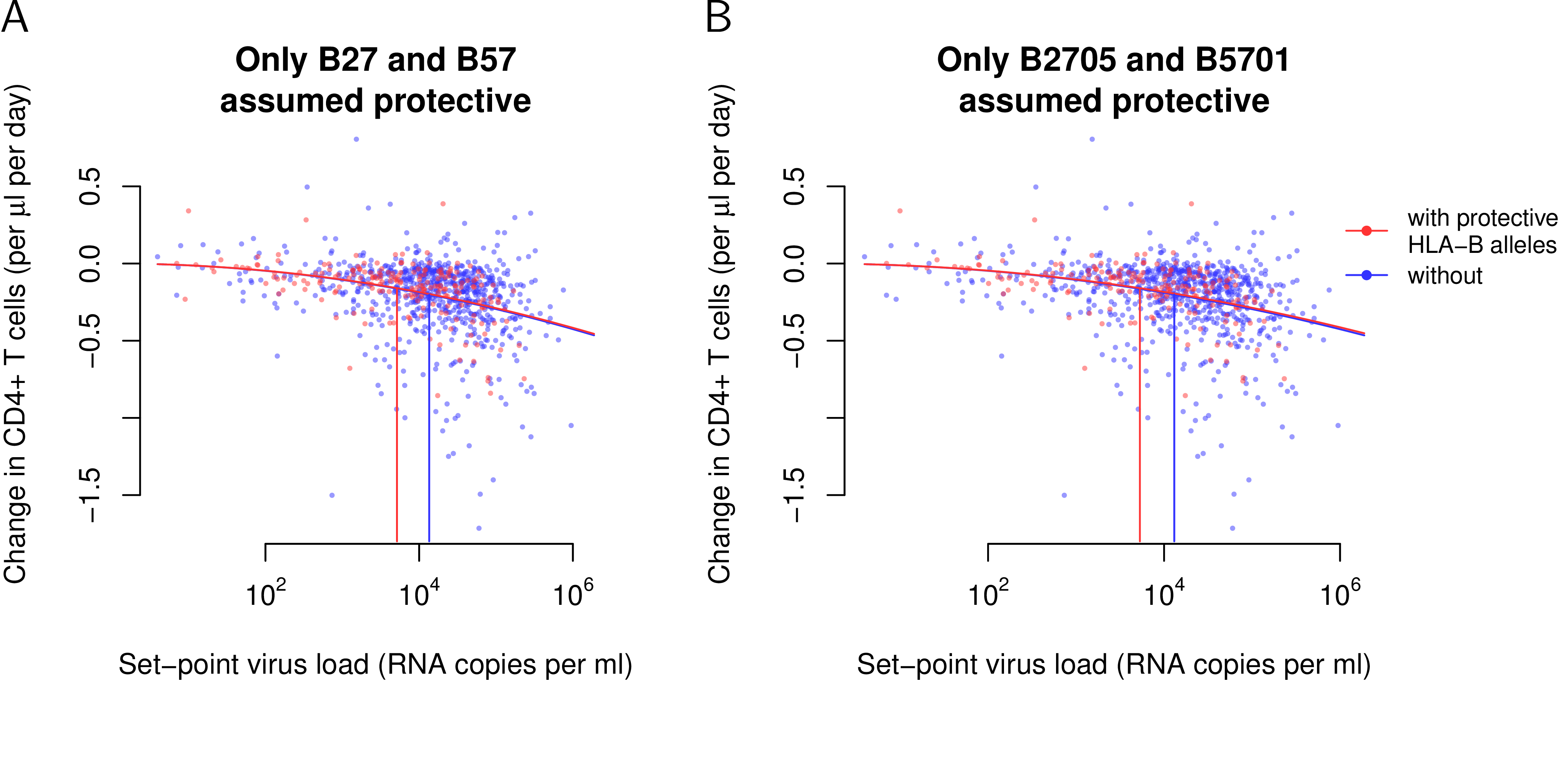

Supplement: Figure S2 — Alternative sets of protective HLA-B alleles and tolerance. (A) Considering only HLA-B27 or 57 as protective, we did not find differences in tolerance between individuals with and without protective HLA-B alleles. (B) We reached the same conclusion if we are even more restrictive and assume only HLA-B*27:05 and *57:01 to be protective. (TIFF) [file pbio.1001951.s002.tiff]

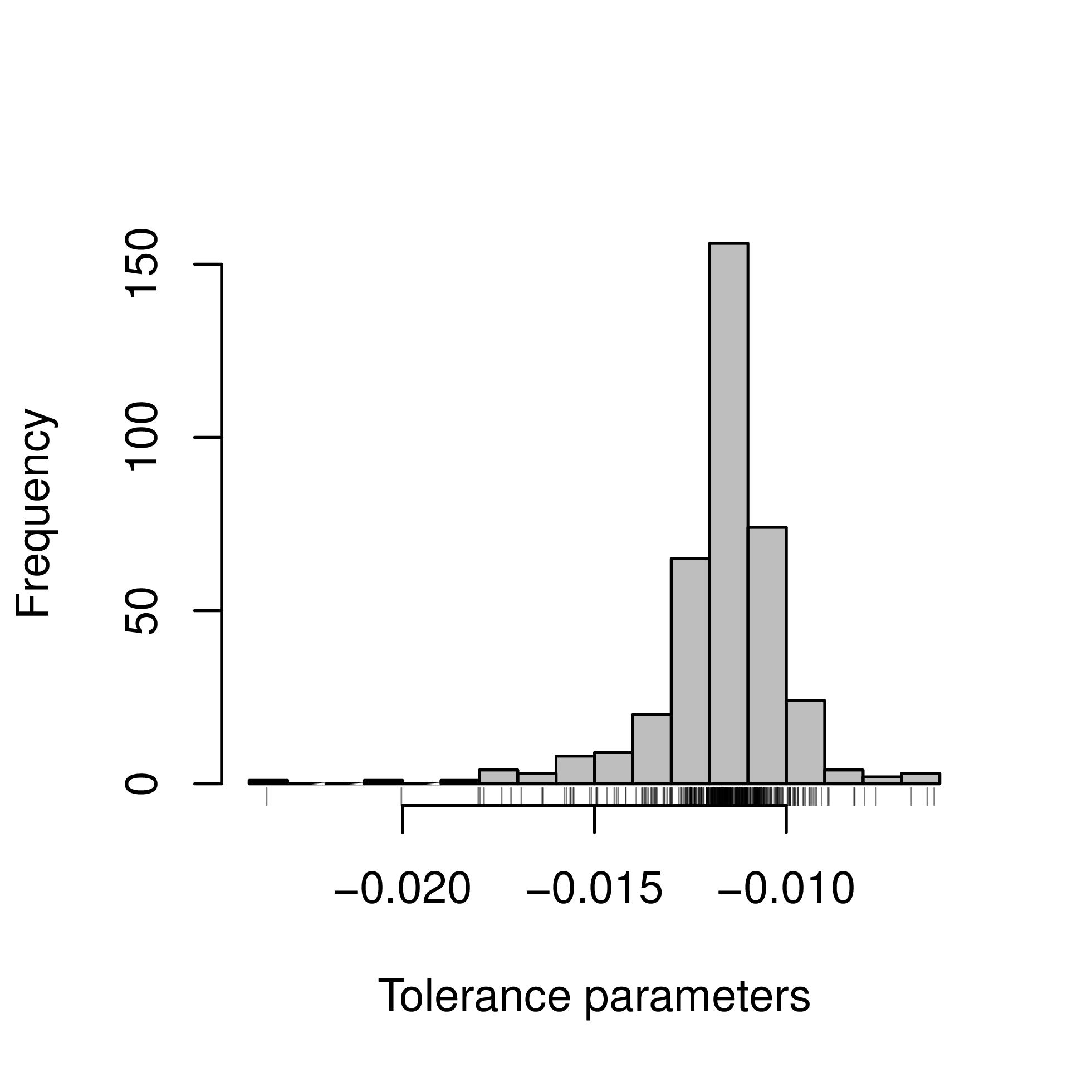

Supplement: Figure S3 — Distribution of the best linear unbiased predictions for the tolerance parameters, , across HLA-B genotypes. (TIFF) [file pbio.1001951.s003.tiff]

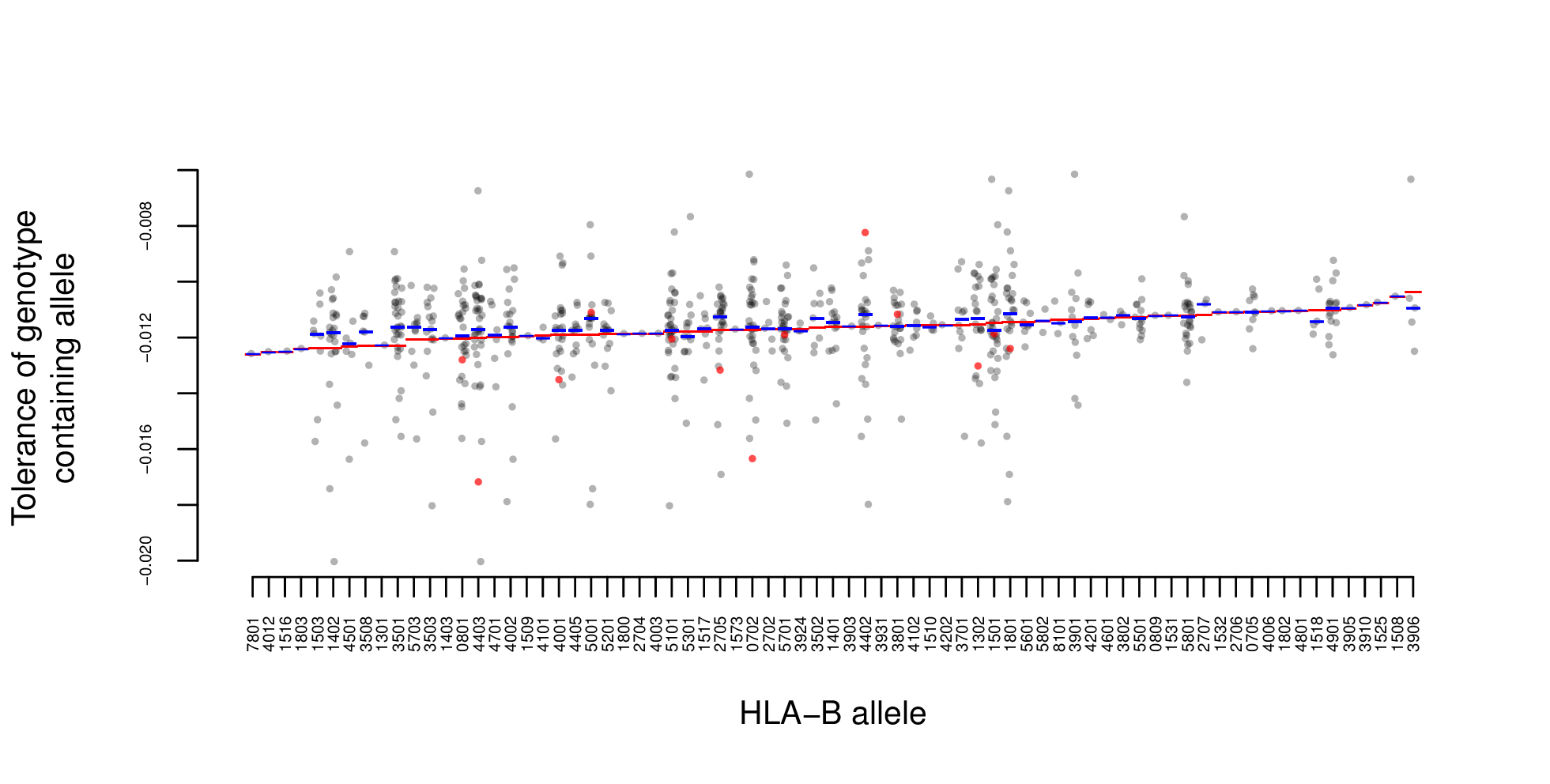

Supplement: Figure S4 — Tolerance by HLA-B allele. The tolerance parameters of genotypes containing an allele are plotted (transparent grey dots). Homozygous genotypes are plotted transparent red. Alleles are ordered by increasing mean tolerance of genotypes that contain the allele (red bars). Blue bars show the median tolerance for each allele. The variation in mean effects of each allele is significantly lower than the tolerance variation across genotypes. (TIFF) [file pbio.1001951.s004.tiff]

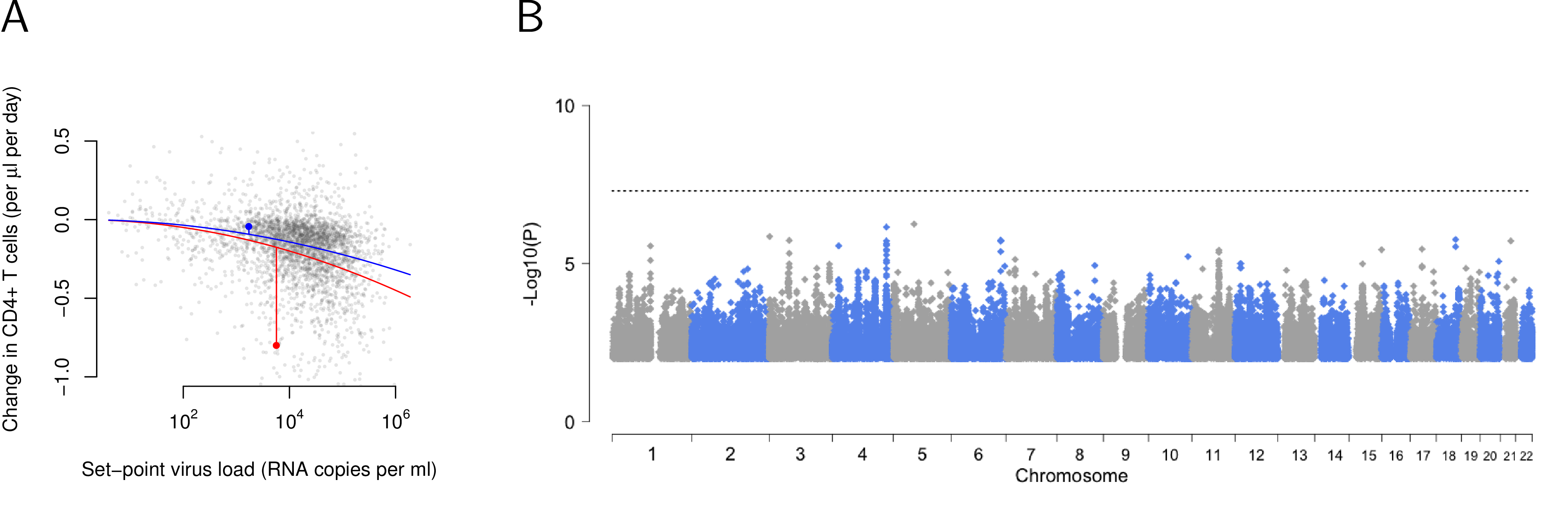

Supplement: Figure S5 — Genome-wide association study. (A) The tolerance phenotype for an individual is defined as the deviation of his/her CD4+ T-cell decline from the average tolerance curve characterizing his/her age class. Two individuals are shown (red and blue dots), together with the tolerance curves (red and blue lines) for people who contract HIV at the same age. In this example, the red and blue individuals contracted HIV at the age of 42 and 20 years, respectively. (B) Manhattan plot showing the p across seven million SNPs. None of the p is above the significance level corrected for multiple testing (dashed line). (TIFF) [file pbio.1001951.s005.tiff]
