# Supplementary material for: Disentangling Human Tolerance and Resistance Against HIV
Source: PLoS Biol. 2014 Sep 16;12(9):e1001951. doi: 10.1371/journal.pbio.1001951 (PMC4165755; doi:10.1371/journal.pbio.1001951)
Supplement: Text S1 — Details on the statistical analyses. This document contains a detailed description of the statistical analyses, the results of which are presented in this article. It also describes additional analyses we performed to corroborate our findings. (PDF) [file pbio.1001951.s007.pdf]

Text S1

Determining the shape of the tolerance curve

We investigated the relationship between CD4+ T cell decline and set-point viral load by a series of regression analyses. A linear relationship $\Delta\text{CD4} = a + b\log_{10} V$, as used for example by Rodrigues et al [1], fits significantly worse than a quadratic relationship $\Delta\text{CD4} = a + b\log_{10} V + c(\log_{10} V)^2$ (F-test: $p = 5.7 \times 10^{-5}$ for the full dataset with 3036 individuals, and $p = 0.015$ for the subset of 923 individuals with *HLA-B* genotype information).

For the full dataset (n=3036), we obtained $a = -0.10 \pm 0.06$, $b = 0.051 \pm 0.032$, and $c = -0.017 \pm 0.004$. For the subset of *HLA-B* genotyped individuals (n=923) we estimated: $a = -0.058 \pm 0.081$, $b = 0.029 \pm 0.046$, and $c = -0.015 \pm 0.006$. However, the linear terms and the intercept are not significantly different from zero.

We therefore based most of the analysis in the present study on the quadratic model: $\Delta\text{CD4} = \alpha(\log_{10} V)^2$ (Equation 1 in the main text). The parameter α in this model measures tolerance (see main text), and is estimated as -0.0111 ± 0.0003 (n=3036), or -0.0117 ± 0.0004 (n=923). The coefficient of determination of the quadratic model fit is 5% (n=3036), or 9% (n=923).

Models including sex and age at infection

We performed a multivariate linear regression of the logarithm of the set-point viral load against sex and age at infection. We found that, on a logarithmic scale to the base 10, the set-point viral load of females is by 0.254 ± 0.035 lower than that of males. This difference was highly significant (p-value=2.2×10^{-13}). We also found that the set-point viral load increased significantly with age at infection (p-value=1.5×10^{-6}). On a logarithmic scale to the base 10, the increase is estimated to be 0.0077 ± 0.0016 per year of age.

We also performed a multivariate linear regression of the CD4+ T cell decline against sex and age at infection. We found no significant association of the CD4+ T cell decline with sex (p-value=0.58). But the CD4+ T cell decline becomes significantly faster with increasing age at infection (p-value=8.2×10^{-13}). With every year of age, the CD4+ T cell slope decreases by 0.0038 ± 0.0005 cells per μl blood per day of infection. For example, for an individual who becomes infected at the age of 20, the CD4+ T cell slope is -0.1428 cells per μl blood per day of infection,

while, for an individual who becomes infected at the age of 21, it is -0.1466 cells per μl blood per day of infection.

The association of tolerance with sex was investigated by fitting the following model:

$$\Delta\text{CD4} = (\alpha_F + \eta_M)(\log_{10} V)^2 \tag{S1}$$

Hereby α_F denotes the tolerance of females, and η_M denotes the offset in tolerance for male individuals, i.e. tolerance in males is $\alpha_F + \eta_M$. The following table shows the parameter estimates of this model:

Parameter	Estimate	Std. Error	t value	Pr(>|t|)
α_F	-0.010941	0.000531	-20.597229	0.000000
η_M	-0.000243	0.000616	-0.393922	0.693666

The parameter η_M, describing the tolerance difference between males and females, is not significantly different from 0. An F-test of this model against the baseline model (Equation 1 in the main text) confirmed this (p-value=0.69).

The relationship between tolerance and age at infection was modeled as a linear effect of age. In particular, we assumed that tolerance is related to the age, a, linearly:

$$\Delta\text{CD4} = (\alpha_0 + c\, a)(\log_{10} V)^2$$

Here α_0 characterizes the tolerance at birth, and c describes the increase or decrease of tolerance per life year. (This equation is identical to Equation 4 in the main text, and is repeated for convenience.) They were estimated as $\alpha_0 = 5.6 \times 10^{-3}$, and $c = -1.6 \times 10^{-4}$/life year. An F-test of this model against the baseline model (Equation 1 in the main text) showed that age at infection has a significant association with tolerance (p-value=10^{-9}).

We combined sex and age at infection into a multivariate analysis by allowing the two parameters in the equation above, α_0 and c, to differ between the sexes:

$$\Delta\text{CD4} = [\alpha_{0,F} + \eta_{0,M} + (c_F + z_M)a](\log_{10} V)^2 \tag{S2}$$

Here, $\alpha_{0,F}$ and c_F denote the tolerance at birth and its change per life years in females. The parameter $\eta_{0,M}$ describes the sex difference of tolerance at birth, and z_M the sex difference between the change of tolerance per life year. We found that neither the tolerance at birth, nor the change of tolerance per life year differ significantly between the sexes as shown in the following table:

Parameter	Estimate	Std. Error	t value	Pr(>|t|)
$\alpha_{0,F}$	-0.005866	0.001726	-3.399015	0.000685*
$\eta_{0,M}$	0.000491	0.002065	0.237836	0.812025
c_F	-0.000167	0.000054	-3.089102	0.002026*
z_M	-0.000001	0.000062	-0.010780	0.991400

Multivariate models including HLA-B homozygosity, carriage of protective HLA-B alleles, CCR5Δ32, and predicted HLA-C expression levels

Carriage of protective *HLA-B* alleles and of *CCR5*Δ32, predicted *HLA-C* expression levels, and *HLA-B* homozygosity were used as covariates in the regression of CD4+ T cell decline against set-point viral load. We did not consider *HLA-B* alleles with detrimental effect as a covariate because they always co-occurred with protective alleles.

We constructed univariate models including each factor in isolation, multivariate models including one of these four factors in combination with sex and age at infection, and a multivariate model including all six covariates. The multivariate model including all six covariates is given by the following equation:

$$\Delta\text{CD4} = [\alpha_0 + \eta_{\text{homo}} + \eta_{\text{prot}} + \eta_{CCR5\Delta32} + \eta_{\text{C-med}} + \eta_{\text{C-hi}} + \eta_{0,M} + (c + z_M)a](\log_{10} V)^2 \quad \text{(S3)}$$

α_0 in this model denotes the tolerance of females at birth, who do not carry protective *HLA-B* alleles and *CCR5*Δ32, and have low predicted *HLA-C* expression. The parameters η_{homo}, η_{prot}, $\eta_{CCR5\Delta32}$, $\eta_{\text{C-med}}$, and $\eta_{\text{C-hi}}$, denote the offsets in tolerance in individuals who are *HLA-B* homozygous, carry protective *HLA-B* alleles, *CCR5*Δ32, or have medium or high *HLA-C* expression, respectively.

The estimates of the parameters of the multivariate regression model (Equation S3) are given in the following table:

	Estimate	Std. Error	t value	Pr(>|t|)
α_0	-0.012842	0.002973	-4.319891	0.000018*
η_{homo}	-0.006707	0.001858	-3.609466	0.000327*
η_{prot}	0.000312	0.000946	0.329876	0.741586
$\eta_{CCR5\Delta32}$	0.000709	0.001163	0.610052	0.542013
$\eta_{\text{C-med}}$	0.001037	0.001022	1.014641	0.310605
$\eta_{\text{C-hi}}$	0.000798	0.001390	0.573747	0.566312
$\eta_{0,M}$	0.003640	0.003473	1.047878	0.295034
c_F	-0.000074	0.000079	-0.942591	0.346195
z_M	0.000002	0.000093	0.024057	0.980814

Of the four factors, only *HLA-B* homozygosity is significantly associated with tolerance. For this smaller dataset, an association of tolerance with the age at infection is not detectable. An *F*-test against a model without *HLA-B* homozygosity as a covariate corroborated that homozygosity significantly associates with tolerance:

	Res.Df	RSS	Df	Sum of Sq	F	Pr(>F)
1	747	36.82				
2	748	37.46	-1	-0.64	13.03	0.0003

To assess if this multivariate regression is confounded by differences in the ethnicity between the individuals in our study population, we excluded individuals who were not of European ancestry. We found that *HLA-B* homozygosity remains

the only significant effect and the parameters estimates change only marginally: $\alpha_0 = -0.0137 \pm 0.0032$, $\eta_{\text{homo}} = -0.0065 \pm 0.0019$. An F-test against a model without homozygosity results in a p-value of 0.0006.

Mixed effects models with combined HLA-B genotype

The potential effect of combined *HLA-B* genotypes on tolerance was included as a random effect:

$$\Delta\text{CD4} = (\overline{\alpha} + \alpha_h)(log_{10}V)^2$$

(This equation is identical to Equation 2 in the main text, and is repeated for convenience.) In this model, we divided the tolerance parameter into an average component $\overline{\alpha}$, and a random effect α_h associated with the *HLA-B* genotype. The random effect was assumed to be normally distributed with mean zero, and standard deviation σ_h. The mixed effects modeling approach estimates this standard deviation σ_h, rather than a specific tolerance parameter for each of the 375 *HLA-B* genotypes. Thus, the mixed effects model has only two additional parameter, $\overline{\alpha}$ and σ_h, instead of 375, reducing the risk of overfitting the data. We estimated a standard deviation of $\sigma_h = 0.0040$ with a 95% confidence interval ranging from 0.0029 to 0.0056. A likelihood ratio test against the baseline model (Equation 1 in the main text) corroborates that this effect is significant:

Model	df	log likelihood	Test	likelihood ratio	p-value
Equation 2	3	69.48			
Equation 1	2	62.56	1 vs 2	13.85	0.00020

Including *HLA-B* genotype as a random effect increases the coefficient of determination, R^2, of the tolerance curve to 25.0%.

Approximately half of the 375 genotype groups are represented by only one individual (see Figure 4A). Excluding these genotypes from our analysis makes our results stronger: the significance of the random effect improves from $p = 0.0002$ to $p = 0.00002$, the deviance of the random effect, a measure of the effects size, increases from 0.0040 to 0.0060, and the coefficient of determination becomes $R^2 = 35\%$. If we restrict our analysis to individuals with European ancestry, the random effect remains significant ($p = 0.0008$) and we estimate the deviance of the random effect as 0.0039 with a confidence interval ranging from 0.0027 to 0.0057. These numbers differ only marginally from those estimated from the full dataset.

Including sex and age at infection (and their interaction) as covariates, we obtained an identical estimate for σ_h: 0.0040 with an 95% confidence interval ranging from 0.0029 to 0.0056, and the random effect is significant (likelihood ratio test: p-value=0.00015). Additionally including *HLA-B* homozygosity, protectiveness of *HLA-B* alleles, carriage of *CCR5*Δ32, and predicted *HLA-C* expression levels as covariates, we estimated $\sigma_h = 0.0031(0.0019 - 0.0051)$. Again, a likelihood ratio test against a model without random effect yields a p-value of 0.015, i.e shows that the random effect is significant. The significant improvement of the mixed effects model fit over the model with all covariates (Equation S3) further shows that the

significance of the random effect is not only due to the association of tolerance with *HLA-B* homozygosity.

The mixed effects model predicts the tolerance parameter, α_h, for each combined *HLA-B* genotype. Figure S3 shows a histogram of the best linear unbiased predictions of α_h across *HLA-B* genotypes. These predictions are over-dispersed, which constitutes a deviation from the normality assumption underlying the mixed-effect model. To identify *HLA-B* alleles that are associated with tolerance, we plotted the best linear unbiased predictions [2] of each *HLA-B* genotype against the *HLA-B* alleles they contain (see Figure S4). The average effect we could assign to a particular *HLA-B* allele ranges only from -0.013 to -0.010, whereas the best linear unbiased predictions of each combined *HLA-B* genotype have a much larger range from -0.024 to -0.006. This suggests that the combined effect of two *HLA-B* alleles is not simply the sum of their individual effects. We tested this more formally, by comparing the variances in tolerance of the combined *HLA-B* genotypes to two-times the variance of the average effects of individual alleles with an F-test. The variances of the effects of the combined genotypes are estimated to be 11.5 times larger than the sum of the effects of individual alleles. This effect is highly significant (p-value= 2×10^{-16}). We also found that homozygous *HLA-B* genotypes had significantly lower best linear unbiased predictions of the tolerance parameter, α_h (Wilcoxon test, $p = 0.002$), consistent with the significant association of tolerance with *HLA-B* homozygosity.

To further investigate if particular *HLA-B* alleles are associated with significantly higher or lower tolerance, we defined a binary factor for each of the 73 alleles in the study population. This factor indicated if an individual carries the *HLA-B* allele in question. A multivariate regression analysis with all the 73 factors showed that none of the 73 *HLA-B* alleles is associated with significantly lower or higher than average tolerance. This finding is consistent with the view that the variation in tolerance associated with the combined *HLA-B* genotypes arises through complex interactions between the two *HLA-B* alleles, rather than just by adding their individual effects. However, it may also be due to a lack of statistical power.

We followed the same procedure for *HLA-A* and *HLA-C*. We found a significant association of tolerance with combined *HLA-C* genotype (p-value=0.026), but none with combined *HLA-A* genotype (p-value=1.00).

Trade-offs between tolerance and resistance

In the context of our tolerance analysis, resistance can be quantified as the average set-point viral load across individuals sharing a genotype. The lower this average set-point viral load, the more resistant the genotype.

To determine the average set-point viral load for each combined *HLA-B* genotype, we regressed the set-point viral load against the combined *HLA-B* genotype, using *HLA-B* genotype as a random effect. The resulting best linear unbiased predictions of the set-point-viral loads are a measure of the level of resistance of each genotype group.

To investigate a potential trade-off between resistance and tolerance, we determined the correlation between best linear unbiased predictions for the set-point viral load and for the tolerance of each combined *HLA-B* genotype. The correlation coef-

ficient did not differ significantly from zero (Pearson's product-moment correlation, p-value=0.40).

References

1. Rodriguez B, Sethi AK, Cheruvu VK, Mackay W, Bosch RJ, et al. (2006) Predictive value of plasma HIV RNA level on rate of CD4 T-cell decline in untreated HIV infection. JAMA 296: 1498-506.

2. Pinheiro JC, Bates DM (2000) Linear mixed-effects models: basic concepts and examples. Springer.
